# Lower-canopy marcescence facilitates light use efficiency of upper-canopy needles in *Cunninghamia lanceolata* in southeast China: implications for plant growth-survival trade-offs

**DOI:** 10.3389/fpls.2025.1681653

**Published:** 2025-10-13

**Authors:** Lili Zhou, Jialong Guo, Zhiguang Zou, Yulong Chen, Qi Liu, Shubin Li

**Affiliations:** ^1^ College of Geography and Oceanography, Minjiang University, Fuzhou, China; ^2^ College of JunCao Science and Ecology, Fujian Agriculture and Forestry University, Fuzhou, China; ^3^ Yingde Forestry Science Research Institute, Qingyuan, China; ^4^ College of Resources and Environment, Fujian Agriculture and Forestry University, Fuzhou, China; ^5^ Forestry College, Fujian Agriculture and Forestry University, Fuzhou, China

**Keywords:** *Cunninghamia lanceolata*, marcescent biomass, light gradient, photosynthetic plasticity, trade-off strategy

## Abstract

Marcescence (the retention of dead leaves) is a widespread trait in many tree species, yet its ecological functions and adaptive significance remain poorly understood. This study examined how four light regimes (open, edge, gap, and interior forests) affect light distribution, marcescent biomass, and upper-canopy photosynthesis in *Cunninghamia lanceolata*, a dominant subtropical timber species in China. We further elucidated its light-optimization mechanisms under low-light conditions. Results revealed that photosynthetic photon flux density (PPFD), irradiance, and transmittance declined from open to interior forests at equivalent canopy heights, while particularly steep declines in PPFD from canopy top to bottom occurred in interior stands (*P* < 0.05). Marcescent biomass was significantly higher in interior forest (3801 g·tree^-1^) and lowest in open stands (1265 g·tree^-1^), with intermediate masses in edge and gap forest. Two-way ANOVA confirmed light regime and canopy height as dominant factors controlling marcescent biomass, with canopy shading promoting upward accumulation in interior forests (*P* < 0.05). Upper-canopy needles in interior forests showed decreased maximum net photosynthetic rate (*P*_max_), light saturation point (LSP), light compensation point (LCP), and dark respiration rate (*R*_d_), but increased light use efficiency (LUE). Lower-canopy marcescent biomass (total, needle, and branch) was significantly negatively correlated with *P*_max_, LSP, LCP and *R*_d_, but significantly positively correlated with leaf-level LUE in the upper canopy (*P* < 0.05), indicating that light limitation-induced marcescence enhances photon use efficiency in upper-canopy needles. These findings highlight an evolutionary growth-survival trade-off strategy in which Chinese fir sacrifices lower-canopy growth to optimize upper-canopy carbon gain under light scarcity, favoring long-term biomass accumulation over short-term growth. By clarifying the functional role of marcescence in low-light adaptation, our study provides globally relevant insights for management of marcescent tree species across different biomes, including density regulation, canopy light optimization, and targeted pruning protocols that leverage retained marcescent biomass for resource allocation efficiency.

## 1 Introduction

Compared to deciduous and evergreen species, marcescent tree species retain their senesced leaves and branches for various time spans from several months to several years. Marcescence is a characteristic trait of certain broadleaved trees, such as *Populus euphratica*, *Quercus robur*, and *Fagus grandiflora* (Angst et al., 2022; Shi et al., 2017), and conifers, such as *Cryptomeria japonica* and *Pseudotsuga menziesii* (Ishii and Kadotani, 2006; Yoshida and Hijii, 2006). In a 33-year-old *Cryptomeria japonica* plantation, canopy-retained dead branches and leaves accounted for 85–90% of total aboveground litter, including both canopy litter and litterfall production (Yoshida and Hijii, 2006). Despite this large biomass retention, the ecological implications of maintaining dead litter aboveground remain insufficiently explored. Studies of *Fagus* and *Quercus* trees and *Calamagrostis epigeios* (a grass which retains a large proportion of plant remains as dead standing biomass) suggest that marcescent leaves typically retain higher nitrogen and exhibit reduced lignin content due to photodegradation. These characteristics facilitate subsequent decomposition and more efficient nutrient release once the leaves are shed (Frouz et al., 2011; Angst et al., 2017), enhancing nutrient uptake by roots in the subsequent growing season (Martins et al., 2021). In *Quercus subpyrenaica*, marcescent leaves contribute to extending the photosynthetic period in transitional forests between temperate and Mediterranean regions, particularly for the significant period of time during September and October (Abadia et al., 1996). However, in temperate ecosystems, marcescent leaf composition is more strongly influenced by precipitation and freeze-thaw cycles than by solar radiation (Brandt et al., 2007). Other reported benefits of marcescence include protection against herbivory (Mingo and Oesterheld, 2009), and a prolonged nutrient retranslocation before leaf abscission (Zhou et al., 2021). However, due to the diversity in plant types and climatic conditions, no consensus has been reached on the causes or ecological significance of marcescence.

Chinese fir (*Cunninghamia lanceolata* (Lamb.) Hook) is an endemic coniferous species widely distributed across southern China, ranging from 21° 31′ to 34° 03′ N, and 101° 30′ to 121° 53′ E (Li et al., 2024). With over 1,000 years of cultivation history, it is the dominant species in forest plantations in China, currently covering 9.90 × 10^6^ ha and with a stand volume of 7.55 × 10^8^ m^3^ (Zhang and Li, 2019). During its long-term evolutionary history, Chinese fir has evolved the trait of retaining senesced branches and needles in the canopy beginning at approximately five years of age, with this marcescent material persisting for several subsequent years (Sheng and Fan, 2002). In young (8-year-old), middle-aged (16-year-old), and mature (25-year-old) stands, canopy marcescent biomass can reach 14.6, 14.2, and 17.4 t ha^−1^, respectively, accounting for 79.1–85.7% of total aboveground litter (Zhou et al., 2021). This retention is strongly influenced by planting density and light availability in Chinese fir plantations. For example, in 4-year-old *C. lanceolata* plantations, increasing stem density from 900 to 6660 stems·ha^−1^ caused the photosynthetic photon flux density (PPFD) in the interior forest to decrease significantly from 99.93 to 10.69 μmol·m^-2^ s^-1^, leading to a dramatic rise in marcescent biomass from 0.76 to 9.84 t·ha^−1^ (Huang et al., 2021). This reduction in PPFD, along with a decreased chlorophyll a/b ratio, accelerates leaf senescence in the lower canopy (Li et al., 2023). Despite previous studies on marcescent biomass distribution, decomposition rates, and nutrient resorption (Zhou et al., 2021), the effects of heterogeneous forest light conditions on marcescent biomass accumulation and the broader ecological implications of marcescence in Chinese fir, remain poorly understood.

Light availability plays a critical role in regulating tree survival and growth, and ultimately ecosystem productivity (Granata et al., 2020). In forest environments, horizontal heterogeneity in light (e.g., open-edge-gap-interior gradients) is accompanied by steep vertical light gradients, with light availability decreasing by over tenfold from canopy top to base (Niinemets, 2007). In lower canopy layers, shading by upper branches restricts light access, thereby reducing the light compensation point (LCP), and dark respiration rate (*R*_d_), and carboxylation efficiency (Sibret et al., 2025; Lamour et al., 2023; Letts et al., 2012). Such conditions also maintain lower photosynthesis rates at saturating light levels and promote chlorophyll degradation, which may induce leaf senescence (Brouwer et al., 2012). Additionally, heterogeneous light environments can impact branch growth and death. For example, Sakhalin spruce branches with a lower length between its basal location and the crown base exhibit a higher probability of death due to reduced PPFD (Chen and Sumida, 2017; 2018). According to the growth–survival trade-off theory, trees prioritize carbon allocation to younger, more productive upper-canopy tissues to maximize light utilization and overall canopy carbon gain (Hartmann et al., 2020; Niinemets et al., 2005).

While canopy photosynthetic acclimation and biomass allocation have been previously investigated (Niinemets, 2010), the physiological consequences of lower-canopy marcescence on upper-canopy photosynthesis remain unclear. Previous evidence indicated that canopy structure and shading strongly influence chlorophyll fluorescence, highlighting how architectural traits regulate light propagation and photosynthetic efficiency in the upper canopy (Balde et al., 2024). In dense stands, reduced light availability can accelerate branch senescence and retention of marcescent biomass in the lower canopy, while simultaneously limiting photosynthetic capacity in upper canopy needles by altering vertical resource distribution (Durand et al., 2022). Alternatively, marcescent tissues may act as light-diffusing elements, redistributing incoming radiation more evenly and thereby enhancing light use efficiency (LUE) of productive foliage under suboptimal light conditions (Plaga et al., 2024). Collectively, these studies suggest that lower-canopy marcescence may not only influence local light microenvironments but also indirectly modulate upper-canopy photosynthesis. Understanding these interactions is therefore crucial for elucidating how light competition and marcescence jointly regulate canopy-level photosynthetic function, particularly in dense plantations.

Previous studies of Chinese fir have documented the spatial and temporal distribution of marcescence across developmental stages, and variations in photosynthetic efficiency across canopy layers, needle age, and planting densities (Zhou et al., 2021; Huang et al., 2021). However, the direct effects of light gradients on marcescent biomass, and the subsequent consequences for upper-canopy photosynthesis, remain largely unexplored. We therefore hypothesized that: (1) horizontal and vertical light gradients differ significantly across open, edge, gap, and interior forest conditions, with reduced light availability in the lower canopy of interior forests promoting increased marcescent biomass and upward redistribution of dead branches and needles; (2) photosynthetic characteristics in upper-canopy needles (e.g., *P*_max_, LSP, LCP) are reduced in interior forest compared to open forest; and (3) lower-canopy marcescence, induced by limited light conditions, may enhance LUE in upper-canopy needles, ultimately improving plant capacity to survive under competitive conditions. The specific objective of this study is to assess how insufficient light-mediated lower-canopy marcescence affects LUE in upper-canopy needles. To test these hypotheses, we measured PPFD, illuminance and transmittance along a natural light gradient spanning open-edge-gap-interior Chinese fir forests. We also quantified the vertical distribution of marcescent biomass and upper-canopy needle photosynthetic parameters across these distinct light gradients. Finally, regression models were developed to link lower-canopy marcescent biomass and upper-canopy needle photosynthetic performance. Our results contribute to a better understanding of the ecological adaptation strategies of marcescent species and provide valuable guidance for optimizing planting densities, thinning, and pruning practices in plantations.

## 2 Material and methods

### 2.1 Study site

**Figure 1:**
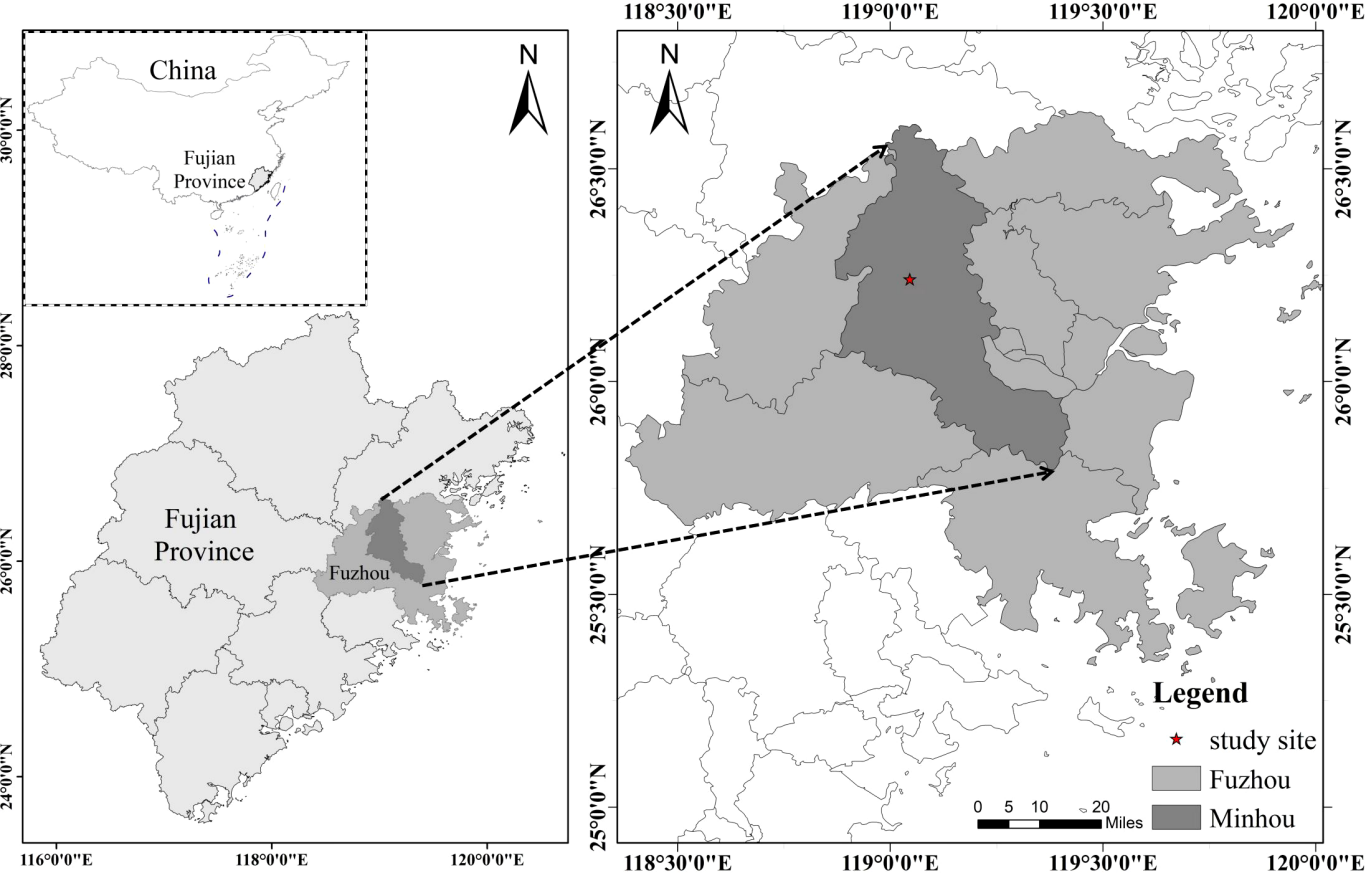
Location of the study site in Baisha National Forest Farm, Minhou County, Fuzhou City, Fujian province, China.

This study was conducted at Baisha National Forest Farm (119° 04′ 32″ E, 26° 24′ 12″ N) in Minhou County, Fuzhou City, Fujian Province, southeastern China (**Figure 1**). The region experiences a subtropical monsoon climate, with a mean annual temperature of 17.15 ± 2.35 °C. Average annual maximum and minimum temperatures are 23.6 °C and 16.4 °C, respectively, with recorded extremes of 40.6 °C and -4 °C. Mean annual precipitation is 1673.9 mm, distributed across approximately 150 rainy days (accounting for 41.8% of total days in a year). The frost-free period spans 240–320 days a year. Soils are classified as mountainous red soils developed from sandstone parent material, with pH 4.02–4.20, bulk density 1.11–1.36 g·cm^-3^, organic matter content 16.3–38.9 g·kg^-1^, total nitrogen 0.51–1.14 g·kg^-1^, total phosphorus 0.08–0.14 g·kg^-1^, and total potassium 3.98–13.4 g·kg^-1^ (Wang, 2008). The forest understory is dominated by shade-tolerant and mesophytic species such as *Pieris multiflora*, *Gardenia jasminoides*, *Cleistocalyx operculatus*, *Liriope* sp*icata*, *Woodwardia japonica*, *Adiantum capillus-veneris*, *Ampelopsis sinica*, and *Carex tristachya*.

The experimental site is located in Compartment 101, Sub-compartment 11-15, of the Gulou Working Area, where a Chinese fir plantation was established in January 2017. The *C. lanceolata* seedlings were from a third-generation seed orchard in the local area to ensure genetic uniformity. The terrain was prepared using horizontal strip bench terracing, and seedlings were planted at a spacing of 1.8 m × 2.0 m. Post-planting management included site cultivation and weeding in the first year, followed by two weeding treatments in the second year and one in the third year to reduce understory competition and promote seedling growth. To evaluate the effects of different light environments on canopy structure and photosynthesis, three permanent plots (20 m × 20 m) were established within the stand at approximately 243 m a.s.l., on southeast-facing slopes with slope angles of ~28°. Plots were established at least 20 m apart to minimize edge effects and ensure independent sampling. All trees within the plots were inventoried, and their diameter at breast height (DBH) and total height were measured. Based on these measurements, representative trees were selected from four typical light environments: open forest (isolated trees without shading), edge forest (transition zone), gap forest (canopy opening), and interior forest (shaded understory). Edge forest was defined as the transition zone between the forest and adjacent open land, extending ~10–20 m inward from the forest boundary, where microclimatic conditions are strongly influenced by lateral light penetration. In each plot, one tree was selected from the open forest, one from the edge, one from the gap, and one from the interior forest, resulting in a total of 12 individual trees across the three replicate plots. The target trees in different light environments were healthy individuals with DBH and height close to the mean and without visible disease or damage. These trees were used to investigate marcescent branch and leaf retention, within-canopy light distribution, and photosynthetic traits to clarify the structural and functional responses of Chinese fir to different light conditions. All the measurements were conducted in September 2023.

### 2.2 Light environment measurements

To characterize vertical marcescent biomass distribution and canopy light environment, the study trees were marked at 0.5 m height intervals using a telescopic pole, beginning from the base of the lowest marcescent branch. In each height layer, the heights of marcescent branch and leaf retention were recorded and photosynthetic photon flux density (PPFD, μmol·m^-2^·s^-1^) was measured with a portable spectroradiometer (HP3500, Taiwan, China) between 09:00 and 11:00 in the morning, during peak daylight hours when light conditions were relatively stable. For each height layer, PPFD was measured in four cardinal directions (SE, NE, SW, and NW) with five replicates per direction, then averaged to improve accuracy. Simultaneously, illuminance was measured at the same points as PPFD using a digital illuminometer (ZDS-10, Shanghai, China) with five replicates per point. Canopy light transmittance (%) was calculated using Equation 1:


(1) $$\text{Transmittance }(\%)=\frac{{\text{PPFD}}_{t\arg et}}{{\text{PPFD}}_{open}}\times 100\%$$

where *PPFD*_target_ is the PPFD measured within the target tree, and *PPFD*_open_ is the PPFD measured in an open field under full sunlight at the same time. Canopy light transmittance reflects the relative availability of light under different canopy conditions and vertical strata.

### 2.3 Marcescent biomass measurements

Marcescent branches and needles were collected from each target tree using pole pruners and manual tree climbing. Dead branches were sequentially pruned from the bottom to the top of the trunk, and from multiple directions. The standard trees were approximately 3.5 m tall and were divided into seven height layers at 50-cm intervals, and the dry biomass of marcescent needles and branches in each layer (g·tree^−1^) was measured. Total marcescent biomass per tree was then calculated as the sum of all layers. For each height layer, dead branches and needles were collected and immediately sealed in plastic bags to prevent moisture loss. Samples were then transported to the laboratory, where they were separated into dead branches and dead needles, and oven-dried at 75 °C to a constant weight. Dry mass was measured with a precision balance (EX125XZH, OHAUS, Parsippany, USA).

### 2.4 Determination of light response curves and photosynthetic parameters

After removal of marcescent biomass, the canopy of each target tree was vertically divided into three layers: upper, middle, and lower. The upper canopy was defined as the portion within 1.5 m from the treetop; the lower canopy was the portion within 1.5 m above the lowest live branch; and the middle canopy represented the remaining intermediate layer. A compass was used to identify the four directions (southeast, northeast, southwest, and northwest). In each canopy layer and direction, the longest branch was selected, and mature, fully expanded needles at the distal end were sampled for photosynthetic measurements. Measurements were taken separately from each direction, and the mean value was used as the photosynthetic indicator for the same layer of the same tree.

Net photosynthetic rate (*P*_n_) was measured using a portable photosynthesis system (CIRAS-3, PP Systems, USA) under standardized conditions: photosynthetically active radiation (PAR) at 1000 μmol·m^-2^·s^-1^ and CO_2_ concentration at 400 μmol·mol^-1^. The temperature inside the CIRAS-3 chamber during gas exchange measurements was maintained at 29 ± 1 °C, corresponding to ambient conditions in September, to minimize temperature-induced variability. September was selected as it represented the late growing season when needles were fully mature and photosynthetic activity remained stable under moderate climatic conditions, avoiding the confounding effects of summer heat stress and winter dormancy. Photosynthetic light response curves were subsequently measured using the automated light program of the CIRAS-3 system. Incident PAR was sequentially adjusted to 1800, 1600, 1400, 1200, 1000, 800, 600, 400, 200, 150, 100, 50, and 0 μmol·m^-2^·s^-1^. At each light level, data were recorded for 2 minutes with automatic calibration between steps. To minimize potential variability, all measurements were performed between 09:00 and 11:00 in the morning under stable light and temperature conditions, and following a consistent protocol across all trees. Photosynthetic measurements were conducted over 11–15 September 2023, with all measurements in each plot completed within two consecutive days to ensure that data were collected during the same continuous period. To estimate photosynthetic parameters, a non-rectangular hyperbola model (Ye et al., 2019) was fitted to the light-response data using Equation 2:


(2) $$P_{n}=\frac{\alpha I+P_{\max}-\sqrt{{\left(\alpha I+P_{\max}\right)}^{2}-4\theta \alpha IP_{\max}}}{2\theta }-R_{\text{d}}$$

Where, *P*_n_ is the net photosynthetic rate (μmol·m^-2^·s^-1^), *I* is the incident PAR (μmol·m^-2^·s^-1^), α is the initial slope of the light response curve (quantum yield), *P_max_* is the maximum net photosynthetic rate, *θ* is the curvature factor (dimensionless, 0 < *θ* ≤ 1), *R*_d_ is the dark respiration rate (μmol·m^-2^·s^-1^). Key photosynthetic parameters derived from the fitted curve included light compensation point (LCP), light saturation point (LSP), apparent quantum yield (AQY), maximum net photosynthetic rate (*P*_max_), and dark respiration rate (*R*_d_).

Leaf-level light use efficiency (LUE) was calculated as the ratio of *P*_n_ to the amount of absorbed PAR at the leaf surface, representing the amount of carbon assimilated per unit of absorbed light energy (Equation 3; Cheng et al., 2023):


(3) $$\text{LUE }(\text{μ}mol\;CO_{2}\cdot \;\mu mol^{-1\;}photons)=\frac{P_{n}}{{\text{PAR}}_{leaf}}=\frac{P_{n}}{\text{PAR}\times \alpha_{leaf}}$$

Where *P*_n_ is the net photosynthetic rate (μmol CO_2_·m^-2^·s^-1^), *PAR*_leaf_ is the absorbed PAR at the leaf level (μmol photons·m^-2^·s^-1^), calculated by multiplying incident PAR by the leaf absorptance (α*_leaf_*). The leaf absorptance is typically assumed to be 0.85 for most green leaves (Niinemets, 2007).

### 2.5 Statistical analysis

Statistical analyses were performed following verification of homogeneity of variances using Levene’s test. When the assumption of homogeneity was violated, data were log_10_ or square-root transformed prior to analysis. Means were compared by Duncan’s multiple range test at significance level of *P* < 0.05. One-way analysis of variance (ANOVA) was used to evaluate differences in measured variables both among different light environments at the same canopy height and among different height strata within the same light environment (*P* < 0.05). Two-way ANOVA was conducted to assess the interactive effects of light regime and canopy height on lower-canopy light conditions, marcescent biomass, and upper-canopy photosynthetic parameters. Pearson correlation analysis was used to examine relationships among all measured variables. Both linear and nonlinear regression models were applied to describe the relationships between upper-canopy photosynthetic parameters and lower-canopy marcescent biomass as defined in Section 2.4. All statistical analyses were performed using Microsoft Excel 2016 and IBM SPSS Statistics 25 (IBM Corp., Armonk, NY, USA). Figures were generated with Origin 2021 (OriginLab Corp., Northampton, MA, USA). Data are presented as mean ± standard deviation (SD).

## 3 Results

### 3.1 Horizontal and vertical light distribution in under-canopy layer across different light regimes

**Figure 2:**
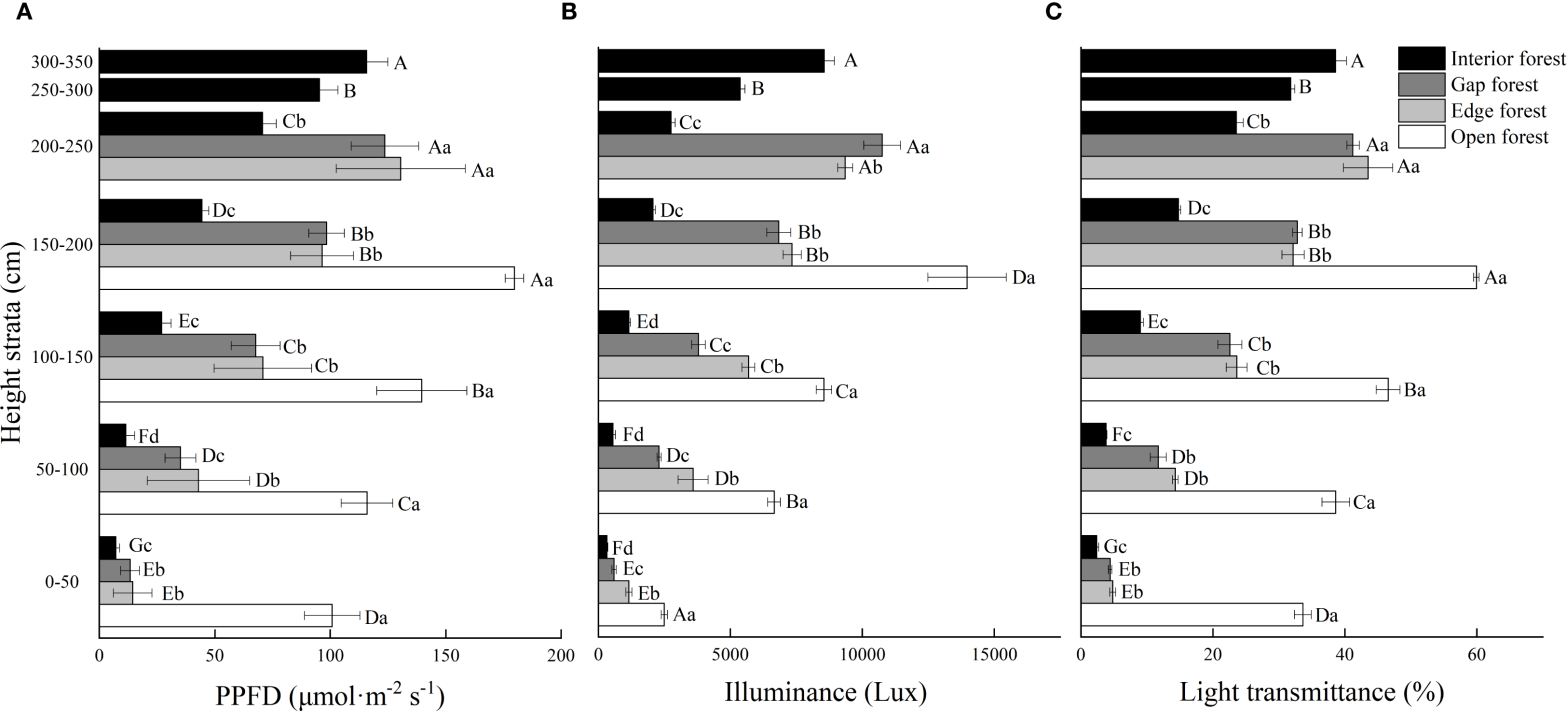
Vertical characteristics of **(A)** PPFD (μmol·m^-2^ s^-1^), **(B)** illuminance (Lux), **(C)** transmittance (%), of Chinese fir trees in different light regimes. Values are means ± standard deviation (n = 3). Different uppercase letters indicate significant differences among different height strata under the same light regime, while different lowercase letters indicate significant differences among different light regimes in the same height strata (*P* < 0.05).

Light regime, height strata and their interaction had significant effects on PPFD, illuminance and transmittance (*P* < 0.001; **Table 1**). Across all four light regimes, PPFD, illuminance, and transmittance increased significantly with increasing height strata (*P* < 0.05), although the magnitude of vertical variation varied considerably among open, edge, gap, and interior forests (**Figure 2**). The greatest vertical gradients were observed in interior forests. For example, PPFD in the highest layer (300–350 cm) of interior forest was 16 times higher than in the lowest layer, whereas in edge and gap forests the corresponding increases were about 9-fold, and in open forests less than 2-fold (**Figure 2A**). Horizontal comparisons at each height revealed that open forests consistently exhibited the greatest PPFD, while interior forests had the smallest. For example, at 0–50 cm, the PPFD of trees in the interior forest was <10% of that in open forest.

**Table 1:** Two-way ANOVA of light regime (df = 3), height strata (df = 6) and their interaction on photosynthetic photon flux density (PPFD), illuminance and transmittance of the target Chinese fir trees.

Factors	PPFD		Illuminance		Transmittance	
	*F*	*P*	*F*	*P*	*F*	*P*
Light regime	1683.558	0.001^***^	646.227	0.001^***^	1683.286	0.001^***^
Height strata	849.720	0.001^***^	500.353	0.001^***^	849.621	0.001^***^
Light regime × Height strata	19.927	0.001^***^	54.655	0.001^***^	19.929	0.001^***^

***p < 0.001.

Patterns of illuminance and transmittance showed similar patterns. Vertical differences in illuminance were most pronounced under lower light regimes, with ratios of the highest to lowest height strata ranging from 5.6-fold in open forest to almost 27-fold in interior forest. The corresponding ratios in edge and gap forests were 8.1 and 18.2 respectively. Horizontal differences in illuminance were greatest in the 150–200 cm height strata (**Figure 2B**). Transmittance was significantly higher in open forest across all height strata. At 150–200 cm, transmittance in edge, gap, and interior forest was only 53.6%, 54.7%, and 24.7% of that in open forest (**Figure 2C**). These patterns indicated that canopy shading intensifies vertical stratification of light, particularly in interior forests, where light availability in the lower canopy is strongly limited.

### 3.2 Horizontal and vertical distribution of under-canopy marcescent biomass across different light regimes

**Figure 3:**
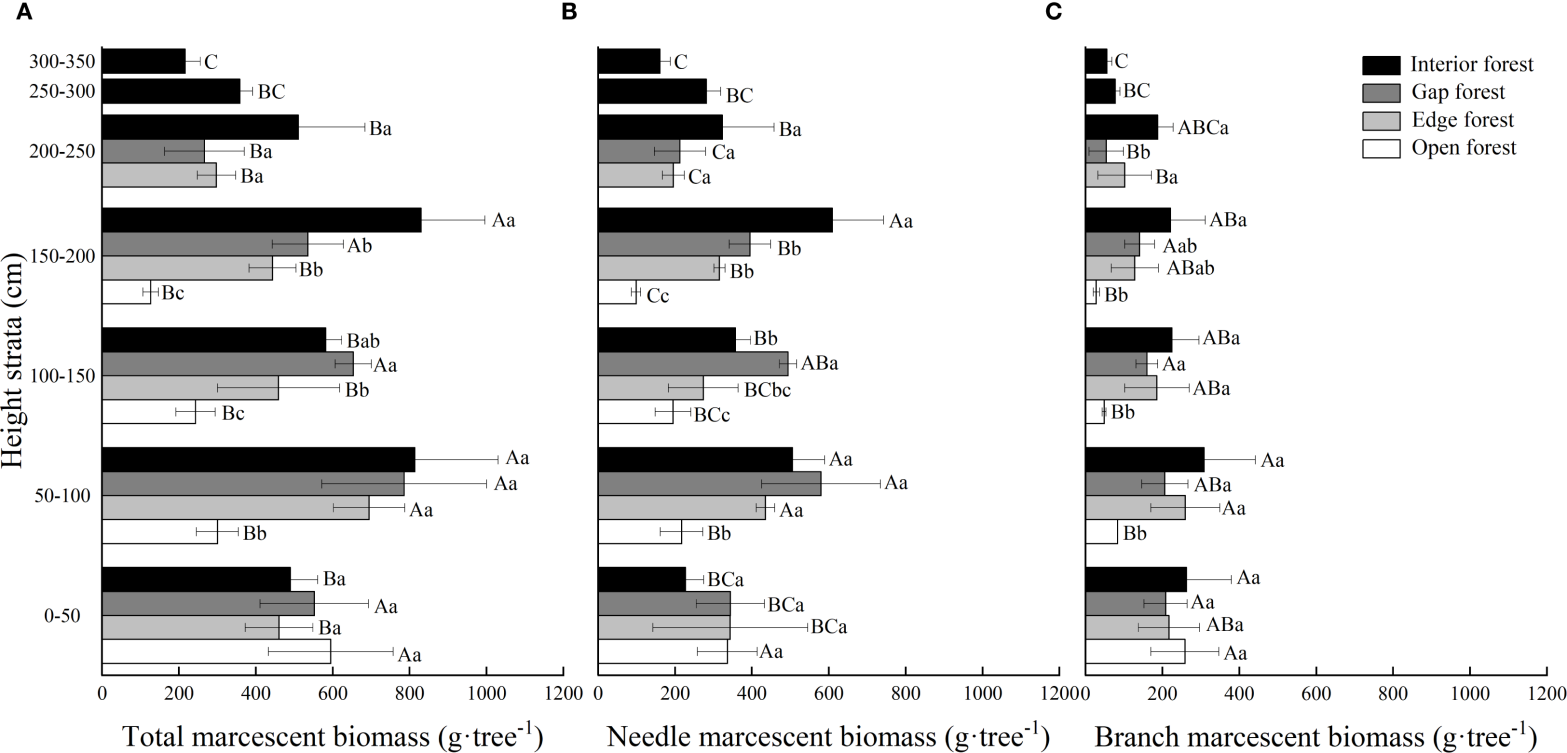
Vertical distribution of marcescent biomass of Chinese fir trees in different light regime. **(A)** Total, **(B)** needle, and **(C)** branch senescent biomass. Values are means ± standard deviation (n=3). Different uppercase letters indicate significant differences among different height strata under the same light regime, while different lowercase letters indicate significant differences among different light regime in the same height strata (*P* < 0.05).

Light regimes, height strata and their interactions significantly affected total, needle, and branch marcescent biomass with the exception of the interaction effect on branch marcescent biomass (*P* < 0.001; **Table 2**). Both the retention height and the total marcescent biomass increased significantly from the open forest to the interior forest. The mean retention height was significantly lowest in open forest (1.97 m) and highest in interior forest (3.42 m), with intermediate values for edge and gap forests. Similarly, the mean total marcescent biomass was significantly lowest in open forest (1265 g·tree^-1^) and highest in interior forest (3801 g·tree^-1^), with edge and gap habitats exhibiting intermediate values (2456 and 2794 g·tree^-1^, respectively) (**Figure 3A**). Moreover, the vertical distribution of marcescence varied markedly among light regimes. In open forest, the majority of marcescent biomass was concentrated at 0–50 cm (47% of total), with decreasing proportions observed in higher strata. In edge and gap habitats, marcescence predominantly occurred at 50–100 cm (about 28% of total). In interior forest, marcescent biomass was concentrated at both 50–100 cm and 150–200 cm, with significantly higher values than in other height strata (**Figure 3A**).

**Table 2:** Two-way ANOVA of light regime (df = 3), height strata (df = 6) and their interaction on marcescent biomass of the target Chinese fir trees.

Factor	Total marcescent biomass		Needle marcescent biomass		Branch marcescent biomass	
	*F*	*P*	*F*	*P*	*F*	*P*
Light regime	24.796	0.001^***^	27.853	0.001^***^	11.758	0.001^***^
Height strata	15.532	0.001^***^	15.055	0.001^***^	10.518	0.001^***^
Light regime × Height strata	4.74	0.001^***^	6.768	0.001^***^	1.320	0.247

***P < 0.001.

Both needle and branch marcescent biomass differed significantly among the four light regimes. On average, needle marcescent biomass increased from 847.0 g·tree^-1^ in open forest to 2466 g·tree^-1^ in interior forest, while the branch marcescent biomass was 417.5 and 1336 g·tree^-1^, respectively (**Figures 3B, C**). In open forest, both needle and branch litter were mainly concentrated in the 0–50 cm height strata, while in edge and gap habitats, they mainly occurred at 50–100 cm. In interior forest, the maximum accumulation of needle marcescent biomass was at 150–200 cm, whereas branch marcescence peaked at 50–100 cm. Notably, at 300–350 cm, needle and branch marcescent biomass were still present in interior forest, but were absent or minimal for trees in the other light regimes.

### 3.3 Photosynthetic characteristics of upper-canopy needles across different light regimes

**Figure 4:**
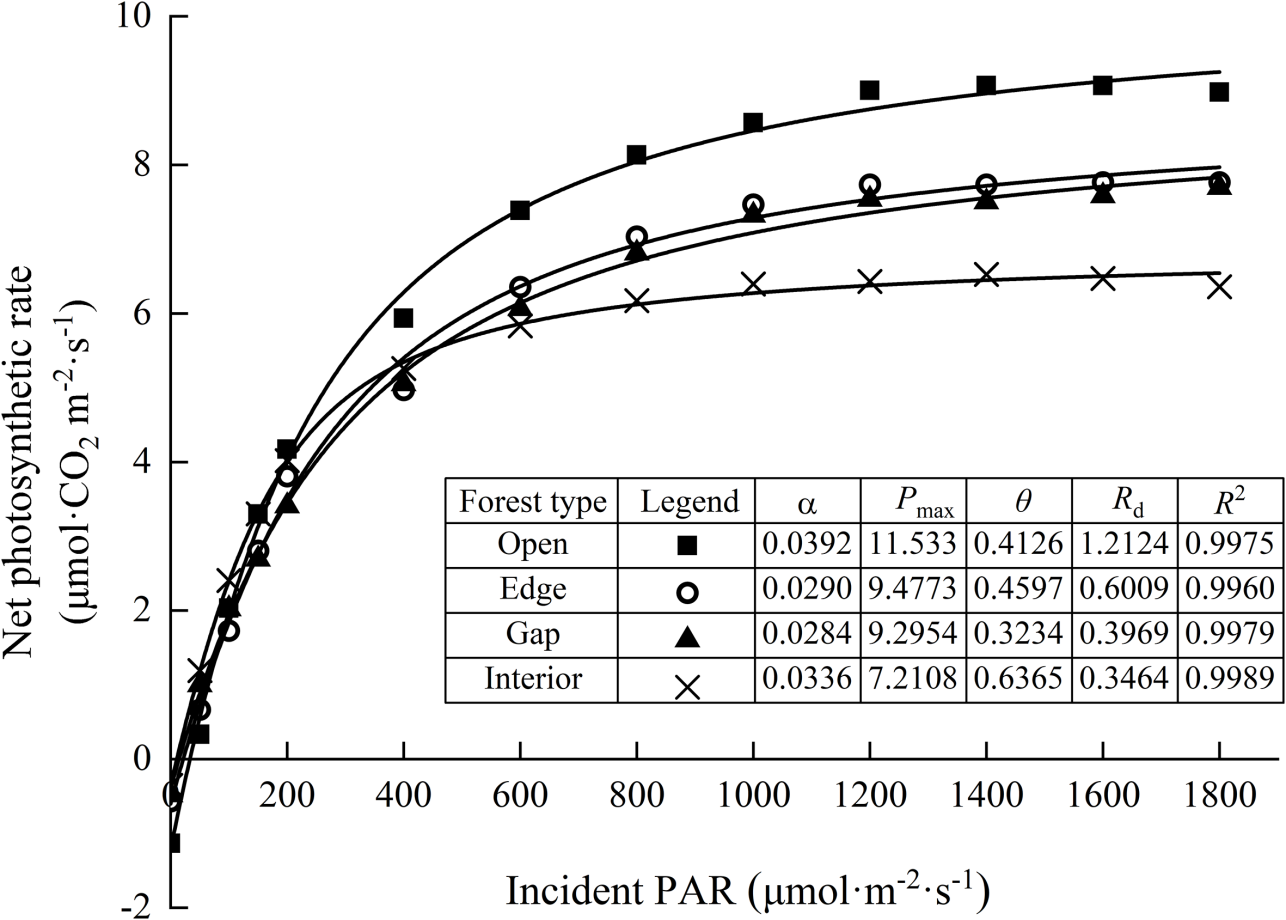
Light response curves of upper-canopy needles of Chinese fir trees among varying light regimes. Values are means ± standard deviation (n=3). α the initial slope of the light response curve, *P*_max_ the maximum net photosynthetic rate, *θ* the curvature factor, *R*_d_ the dark respiration rate, *R* ^2^ goodness of fit.

Based on the fitted light response curves (**Figure 4**), the net photosynthetic rate of needles increased almost linearly under low light intensities (0–200 μmol·m^−2^;·s^−1^) in all light regimes, with no significant differences observed. At moderate light intensities (200–1000 μmol·m^−2^;·s^−1^), the rate of increase in photosynthesis began to slow. When light intensity exceeded 1000 μmol·m^−2^;·s^−1^, interior-forest needles reached light saturation first, with their net photosynthetic rate plateauing. In contrast, needles in edge and gap habitats continued to show a slight increasing trend, whereas those in the open habitat exhibited a notable increase, indicating they had not yet reached saturation. Under high light intensities (>1400 μmol·m^−2^;·s^−1^), a slight photoinhibition effect was observed in both interior and open forest habitats, as evidenced by a minor decline in net photosynthetic rate.

Two-way ANOVA indicated that light regime significantly affected AQY, *P*_max_, LSP, *R*_d_ and LUE (*P* < 0.05), and canopy layer (as defined in Section 2.4) significantly influenced *P*_max_, LCP and *R*_d_. However, there was no significant interaction effect between light regime and canopy height on any photosynthetic parameter (**Table 3**). Photosynthetic parameters were estimated by fitting light response curves using the rectangular hyperbola model (**Table 4**). The AQY ranged from 0.015 to 0.051 mol·mol^-1^, with no significant differences among the light regimes. The *P*_max_ and LSP were significantly highest in the open forest and lowest in the interior forest, with intermediate values in the edge and gap habitats, reflecting the varying light adaptation capacities of Chinese fir needles. Similar trends were observed for the light compensation point (LCP) and dark respiration rate (Rd). In contrast, the LUE was significantly enhanced in the interior forest (0.024) compared to the open forest (0.006), indicating a superior ability of interior forest canopies to utilize light energy under low-light conditions.

**Table 3:** Two-way ANOVA of light regimes (df = 3), canopy layer (df = 2), and their interaction on leaf photosynthetic parameters of the target Chinese fir trees.

Factor	AQY		*P*_max_		LSP		LCP		*R*_d_		LUE	
	*F*	*P*	*F*	*P*	*F*	*P*	*F*	*P*	*F*	*P*	*F*	*P*
Light regime	5.562	0.001^***^	7.133	0.001^***^	11.86	0.001^***^	2.237	0.087	3.567	0.016^*^	68.697	0.001^***^
Canopy layer	0.106	0.899	3.306	0.04^*^	0.302	0.74	4.387	0.014^*^	3.822	0.024^*^	1.071	0.346
Light regime × Height strata	0.48	0.822	1.064	0.388	0.395	0.881	0.193	0.978	0.467	0.832	0.481	0.821

*P < 0.05; ***P < 0.001. AQY apparent quantum yield, *P*_max_ maximum net photosynthetic rate, LSP light saturation point, LCP light compensation point, *R*_d_ dark respiration rate, LUE light use efficiency. The definition and measurement of the photosynthetic parameters are contained in Section 2.4, along with the definition of canopy layer (upper, middle, lower).

**Table 4:** Comparison of leaf photosynthetic parameters in the upper canopy of Chinese fir target trees in different light regimes.

Forest type	AQY	*P*_max_	LSP	LCP	*R*_d_	LUE
Open forest	0.043 ± 0.007a	9.10 ± 0.23a	1828.66 ± 8.35a	32.25 ± 6.33a	1.23 ± 0.07a	0.006 ± 0.001c
Edge forest	0.026 ± 0.007a	7.70 ± 0.10b	1733.32 ± 19.53a	18.46 ± 4.23b	0.59 ± 0.12b	0.016 ± 0.002b
Gap forest	0.031 ± 0.016a	7.67 ± 0.56b	1749.76 ± 15.53a	14.51 ± 1.68b	0.42 ± 0.17b	0.013 ± 0.002b
Interior forest	0.045 ± 0.006a	6.16 ± 0.51c	1179.64 ± 222.75b	10.71 ± 2.16b	0.45 ± 0.03b	0.024 ± 0.005a

The definition and measurement of the photosynthetic parameters are contained in Section 2.4. Values are means ± standard deviation (n=3).

Different lowercase letters in the same column indicate significant difference among different light regimes (*P* < 0.05).

### 3.4 Relationships between lower-canopy marcescence and upper-canopy photosynthesis

Correlation analysis revealed that the needle, branch, and total marcescent biomass in the lower canopy (the portion within 1.5 m above the lowest live branch) were significantly negatively correlated with *P*_max_, LSP, LCP, and *R*_d_ of the upper canopy needles (*P* < 0.05), but strongly positively correlated with LUE (*P* < 0.01) (**Table 5**). Significant correlations were also observed among the photosynthetic parameters themselves. *P*_max_ was highly positively correlated with LSP, LCP, and *R*_d_; LCP were significantly positively correlated with LSP and *R*_d_. However, LUE was highly negatively correlated with all four parameters.

**Table 5:** Correlation coefficients between photosynthetic traits of upper-canopy needles in *Cunninghamia lanceolata* and lower-canopy marcescent biomass across all target trees (n = 12) in the different light regimes.

Indicators	AQY	*P*_max_	LSP	LCP	*R*_d_	LUE	Needle marcescent biomass	Branch marcescent biomass
*P*_max_	0.046							
LSP	-0.266	0.876^**^						
LCP	0.023	0.774^**^	0.577^*^					
*R*_d_	0.365	0.771^**^	0.457	0.888^**^				
LUE	0.175	-0.832^**^	-0.730^**^	-0.806^**^	-0.676^*^			
Needle marcescent biomass	-0.023	-0.875^**^	-0.707^*^	-0.894^**^	-0.884^**^	0.851^**^		
Branch marcescent biomass	-0.025	-0.920^**^	-0.875^**^	-0.751^**^	-0.672^*^	0.806^**^	0.826^**^	
Total marcescent biomass	-0.025	-0.931^**^	-0.803^**^	-0.878^**^	-0.840^**^	0.871^**^	0.976^**^	0.929^**^

*P < 0.05; **P < 0.01. AQY apparent quantum yield, *P*_max_ maximum net photosynthetic rate, LSP light saturation point, LCP light compensation point, *R*_d_ dark respiration rate, LUE light use efficiency.

Regression analysis further revealed strong quantitative relationships between upper-canopy photosynthetic parameters and lower-canopy marcescent biomass (**Table 6**). Specifically, *P*_max_ was significantly negative related to needle, branch, and total marcescent biomass. LSP exhibited a quadratic relationship with all three measures of marcescent biomass. LCP had a highly significant linear relationship with both needle and branch marcescent biomass, and an exponential relationship with total marcescence. *R*_d_ was best described by binary or ternary polynomial models. Similarly, LUE exhibited a ternary polynomial relationship with needle and branch marcescent biomass, and an exponential relationship with total marcescent biomass.

**Table 6:** Regression equations between canopy photosynthetic index (Y) and lower-canopy marcescent biomass (X).

Marcescent biomass category	Photosynthetic index	Equation	*R* ^2^	*P*
Needle marcescent biomass	*P*_max_	Y = 10.350 - 0.002 X	0.742	*P<*0.01
	LUE	Y = 10.102X^3^ - 17.711X^2^ + 8.671 X - 0.04	0.807	*P<*0.01
	LSP	Y = 1485.516 + 0.652 X - 0.0003 X^2^	0.575	*P<*0.01
	LCP	Y = 41.158 - 0.013 X	0.780	*P<*0.01
	*R*_d_	Y = 1.699 X^3^ - 0.875 X^2^ - 0.462 X + 2.016	0.873	*P<*0.01
Branch marcescent biomass	*P*_max_	Y = 22.606 - 2.246 ln X	0.833	*P<*0.05
	LUE	Y = 9.345 X^2^ - 6.320X^3^ – 2.446X - 0.012	0.835	*P<*0.01
	LSP	Y = 1743.223 + 0.445 X - 0.0006 X^2^	0.831	*P<*0.01
	LCP	Y = 3849.967 X - 0.813	0.674	*P<*0.01
	*R*_d_	Y = 10.595 X^2^-4.610 X^3^-6.739 X + 2.991	0.707	*P<*0.01
Total marcescent biomass	*P*_max_	Y = 10.462 – 0.001 X	0.852	*P<*0.01
	LUE	Y = e ^-5.607 + 0.907X^	0.822	*P<*0.01
	LSP	Y = 1380.303 + 0.548 X - 0.0002 X^2^	0.846	*P<*0.01
	LCP	Y = 51.989 e ^-0.0004X^	0.838	*P<*0.01
	*R*_d_	Y = 2.384 X^2^-3.186 X + 2.389	0.858	*P<*0.01

See **Table 5** for explanation of the abbreviations.

## 4 Discussion

### 4.1 Light limitation drives marcescence accumulation and vertical redistribution in shaded canopy strata

The results support our first hypothesis that progressive light attenuation from open stands to forest edges, gaps, and interiors leads to significant accumulation of lower-canopy marcescent biomass. Light availability consistently decreased along this gradient, with forest interiors receiving substantially less light than open forests, highlighting strong vertical and horizontal stratification (**Figure 2**). This pattern of horizontal light heterogeneity across different light habitats is consistent with previous studies. For instance, Chondol et al. (2024) reported markedly lower relative photosynthetically active radiation (PAR) in forest interiors than in gaps within broad-leaved Korean pine forests. Simultaneously, vertical light heterogeneity also differed significantly between the light regimes in our study, in the order: forest interior > forest gap > forest edge > open canopy. The PPFD in the highest canopy strata in each habitat was 16.3, 9.31, 9.05, and 1.78 times higher than that at the lowest layer, respectively (**Figure 2A**). The pronounced vertical gradient observed in forest interiors corresponded spatially with the greatest accumulation of marcescent litter. We propose that chronic light deficit accelerated cellular senescence while suppressing abscission-layer formation, a dual physiological response that directly links light stress to marcescent biomass retention.

The mean total marcescent biomass varied significantly among light habitats, peaking in forest interiors (3801 g·tree^-1^) and declining in gaps (2794 g·tree^-1^), forest edge (2456 g·tree^-1^), and open forest (1265 g·tree^-1^) (**Figure 3A**). The nearly 3-fold differential between interior and open forest mirrored the light heterogeneity. Vertical redistribution occurred alongside biomass accumulation, with the highest senescent biomass reaching 3.42 m above the ground in interior forests, compared with 1.97 m in open forests (**Figure 3**), indicating an upward shift in marcescence retention under light-limited conditions, thereby further supporting our hypothesis 1. These findings demonstrate that light regimes regulate not only the onset of leaf senescence and abscission inhibition but also the spatial distribution of litter retention within the canopy. As reported by Lusk (2002), canopy leaves exhibit gradient-dependent divergence in light interception, absorption, and reflection. Sub-compensation-point irradiance with <15 μmol·m^-2^·s^-1^ PPFD in the lower canopy, triggers changes in ethylene and auxin signaling pathway, severely limiting carbon gain (Vandenbussche et al., 2003). This culminates in programmed cell death without abscission-layer activation, manifesting as leaf marcescence rather than abscission (Hossain and Olson, 2023). However, the hormonal basis of light-induced marcescence remains to be conclusively verified.

Marcescence in Chinese fir appears to be an active adaptive strategy with significant ecological implications, especially under shaded forest interior conditions. By prolonging retention of senesced needles and branches in the canopy, Chinese fir enhances nutrient resorption efficiency prior to leaf abscission, thereby optimizing internal nutrient utilization (Wang et al., 2024; Zhou et al., 2021). Furthermore, marcescence helps maintain a high leaf area index (LAI), which supports sustained photosynthetic capacity under low-light conditions (Givnish, 2002). As Hossain and Olson (2023) suggested, the extended retention of senesced foliage in shade-tolerant species promotes continued nutrient acquisition and helps maintain carbon balance in light-limited environments. This strategy stands in sharp contrast to that of light-demanding species, which typically exhibit shorter leaf lifespans and rapid abscission under reduced irradiance (Hansen et al., 2002). Therefore, in evergreen conifers like Chinese fir, marcescence enhances ecological competitiveness by promoting nutrient conservation and carbon balance optimization (Abadia et al., 1996).

### 4.2 Photosynthetic plasticity in response to forest light heterogeneity

Our results support the second hypothesis that upper-canopy photosynthetic traits of *C. lanceolata* vary significantly across light regimes. Upper-canopy needle photosynthetic parameters (*P*_max_, LSP, LCP, and *R*_d_) were significantly lower in interior forest conditions, highlighting reduced photosynthetic capacity under shaded conditions (**Table 4**). This observation is consistent with established principles of photosynthetic acclimation that trees tend to optimize light energy capture and elevate *P*_max_ under high irradiance, while shaded environments constrain photochemical potential due to reduced PPFD. Shade-adapted species typically exhibit lower LSP and LCP to maintain function under low-light conditions (Givnish et al., 2004). Light response curve analyses by Calzavara et al. (2019) further confirmed that shading limits photosynthetic capacity and increases the risk of negative carbon balance. To counteract this, plants may increase chlorophyll content, develop thinner leaves, and reduce respiration to conserve energy (Rozendaal et al., 2006).

In interior forests, *C. lanceolata* adopts a conservative carbon-use strategy to optimize survival under low-light conditions. As a facultative shade-tolerant species, it displays ontogenetic shifts from shade tolerance at the seedling stage to increasing light demand during maturation (Cao et al., 2002). In our study, the upper canopy in interior forests showed lower *P*_max_, LSP, LCP, and *R*_d_, but elevated light use efficiency (LUE), indicating a conservative carbon-use strategy under low-light conditions (Valladares and Niinemets, 2008). These changes suggest a conservative carbon economy that minimizes metabolic costs while maintaining a positive carbon balance, which is essential for long-term survival in light-limited environments (Niinemets, 2023). Similarly, Terashima et al. (2011) highlighted that shade-tolerant species tend to adopt a conservative strategy under low-light conditions—favoring sustained function through reduced respiratory expenditure and higher carbon gain per photon, rather than maximizing photosynthesis. In contrast, light-demanding species typically invest in thicker leaves, elevated chlorophyll a/b ratios, and increased photosynthetic potential under full sunlight (Poorter et al., 2009). However, under shaded conditions, this high-cost strategy may result in a negative carbon budget, limiting growth and survival (Ma et al., 2015). In *C. lanceolata*, reduced *P*_max_ in low-light habitats may hinder short-term growth but supports long-term carbon balance and persistence, demonstrating a classic trade-off between growth and survival. This pattern aligns with the model of Sterck et al. (2006) that links variation in photosynthetic traits to differing growth trajectories across light environments.

### 4.3 Marcescence as an adaptive strategy for optimizing light use efficiency in shaded forests

Our findings support the third hypothesis that the accumulation of lower-canopy marcescent biomass under low-light conditions enhances upper-canopy LUE, providing an adaptive advantage to Chinese fir. Although lower-canopy marcescent biomass was significantly negatively correlated with *P*_max,_ LSP, and LCP, it showed a strong positive correlation with upper-canopy LUE (*P* < 0.01; **Table 5**). These findings suggest that non-photosynthetic tissues may act as biophysical regulators by scattering incident light, enhancing diffuse radiation, and mitigating photoinhibition in shade-adapted leaves. Rezai et al. (2018) demonstrated that diffuse light maintains higher photosystem II (PSII) quantum efficiency under low irradiance, thereby improving LUE, which were consistent with our results. At high-irradiance open-canopy sites, minimal marcescent biomass coincided with reduced LUE, likely due to excess light stress. In contrast, trees in shaded interior forests retained more marcescent biomass and exhibited significantly higher LUE, implying a form of adaptive optimization to light limitation.

Persistent lower-canopy marcescence in shaded environments contributes to energy conservation and supports long-term biomass accumulation in *C. lanceolata.* Additionally, Cheng et al. (2023) demonstrated that persistent dead leaves and branches in shaded understories reduce respiratory carbon loss by extending the functional lifespan of upper-canopy foliage. This supports a growth-survival trade-off strategy, in which energy is allocated to prioritize survival over new growth under resource limitation situations. Similarly, Gravel et al. (2010) proposed that under light scarcity, plants favor the maintenance of existing foliage rather than investing in new leaves, optimizing energy and nutrient allocation. Chen et al. (2020) also reported that closed-canopy species retain more lower-canopy foliage, reduce leaf turnover, and improve nutrient conservation, potentially enhancing canopy-level light capture and long-term productivity.

From an ecological perspective, marcescent litter should not be viewed as a passive by-product of shade stress, but as a functional component of an adaptive canopy structure. By modulating within-canopy light distribution and enhancing upper-canopy LUE, marcescent tissues enable a resource-conserving physiological strategy that sustains carbon gain and promotes survival under shaded conditions. Marcescence may represent an evolutionary innovation in canopy architecture, functioning as an ecological insurance mechanism that converts structural biomass into a persistent light-harvesting scaffold.

From a silvicultural perspective, our findings suggest that strategic retention of lower-canopy marcescent biomass can be incorporated into forest management to enhance canopy-level LUE and carbon resilience. Practices such as selective pruning or stem density adjustments should consider potential trade-offs, for example, excessive removal of marcescent tissues could temporarily reduce harvestable wood yield. Moreover, as stands age, accumulation of lower-canopy marcescent biomass may alter vertical light gradients and photosynthetic responses in upper-canopy foliage, suggesting that management strategies should be adapted to stand development stages. Future research should quantify the relationship between marcescence and LUE across species and climatic gradients to improve predictive accuracy of subtropical plantation management models.

## 5 Conclusion

This study demonstrates that *Cunninghamia lanceolata* adopts marcescence as an adaptive carbon allocation strategy to enhance light use efficiency under low-light conditions. Vertical light heterogeneity in interior forests drives the upward redistribution of marcescent biomass, with canopy height and light availability identified as the primary controlling factors. Under shaded conditions, *C. lanceolata* exhibits a physiological trade-off strategy by suppressing lower-canopy productivity—evidenced by the accumulation of marcescent litter—while significantly enhancing light use efficiency (LUE) in upper-canopy needles. These findings reveal that marcescence serves not merely as delayed litterfall but as an active functional light-regulation mechanism that promotes long-term carbon gain at the expense of short-term growth. This challenges conventional perceptions of canopy litter and provides new insights into adaptive canopy architecture in conifers. From a silvicultural perspective, we recommend maintaining moderate stand densities and applying precision pruning practices that retain an appropriate amount of lower-canopy marcescent biomass, which can maximize physiological benefits by enhancing upper-canopy LUE. Overall, this study provides both ecological and practical guidance for improving carbon efficiency and sustainable productivity in subtropical conifer plantations.

## Data Availability

The raw data supporting the conclusions of this article will be made available by the authors, without undue reservation.
